# Determinants of public health expenditure in the EU

**DOI:** 10.1371/journal.pone.0299359

**Published:** 2024-03-06

**Authors:** Joseph Piscopo, Wim Groot, Milena Pavlova

**Affiliations:** Faculty of Health, Medicine and Life Sciences, Department of Health Services Research, CAPHRI, Maastricht University Medical Center, Maastricht University, Maastricht, The Netherlands; University of Naples - Parthenope: Universita degli Studi di Napoli Parthenope, ITALY

## Abstract

**Background:**

Public health expenditure is one of the fastest-growing spending items in EU member states. As the population ages and wealth increases, governments allocate more resources to their health systems. In view of this, the aim of this study is to identify the key determinants of public health expenditure in the EU member states.

**Methods:**

This study is based on macro-level EU panel data covering the period from 2000 to 2018. The association between explanatory variables and public health expenditure is analyzed by applying both static and dynamic econometric modeling.

**Results:**

Although GDP and out-of-pocket health expenditure are identified as the key drivers of public health expenditure, there are other variables, such as health system characteristics, with a statistically significant association with expenditure. Other variables, such as election year and the level of public debt, result to exert only a modest influence on the level of public health expenditure. Results also indicate that the aging of the population, political ideologies of governments and citizens’ expectations, appear to be statistically insignificant.

**Conclusion:**

Since increases in public health expenditure in EU member states are mainly triggered by GDP increases, it is expected that differences in PHE per capita across member states will persist and, consequently, making it more difficult to attain the health equity sustainable development goal. Thus, measures to reduce EU economic inequalities, will ultimately result in reducing disparities in public health expenditures across member states.

## 1 Introduction

Public health expenditure (PHE) is rapidly increasing in the European Union (EU). The rate of PHE as percentage of Gross Domestic Product (GDP) increased from 6.1% to 7% between 2000 and 2018, peaking at 7.3% in 2009 [1]. In 2018, PHE accounted for the second-largest share of government expenditure in EU member states [2]. Since increases in PHE are not always backed by stronger economic growth, they can impose a burden on public finances.

The COVID-19 pandemic exerted pressure on PHE and led to increases in spending to combat the virus, while economies shrunk. Once the effects of the pandemic were under control, health systems had to face long waiting lists for health services that had been postponed. The high inflationary environment in the post-COVID, Russia’s war on Ukraine and the energy crisis presented further unprecedented challenges to the health sector. In such deteriorating macroeconomic environment, governments are challenged to sustain their health budget and to improve the resilience of public health systems.

In 2015, the governments of the world committed to 17 Sustainable Development Goals (SDGs). SDG 3 presents the health goal, with the aim being to ensure healthy lives and promote well-being for all at all ages by year 2030. This overall health goal has been broken down into 13 targets, with target 3.8, being to ‘achieve universal health coverage, including financial risk protection, access to quality essential healthcare services and access to safe, effective, quality and affordable essential medicines and vaccines for all’. The achievement of this target is measured against two indicators, namely 3.8.1, which measures the universal health coverage and 3.8.2, which measures the proportion of the population with out-of-pocket (OOP) health expenditure amounting to greater than 10% and 25%, as a share of total household expenditure or income.

The high level of OOP health expenditure in particular countries in South and East Europe, is making it more challenging for these countries to achieve the SDG 3 goals. In year 2020, in the EU member states, between 1.0% and 19.2% of households, experienced catastrophic spending on health, meaning the health expenditure forked out of personal funding exceeded 40% of a household’s disposable income [2]. Cylus et al. argue that these figures are underestimating the financial hardship of poor people, whilst overestimating the hardship of the rich ones [3]. The reasons for the high level of OOP health expenditure can be attributed to gaps in coverage, formal payments especially for medicines and, informal payments [4]. Tracking health expenditure and identifying the drivers of this expenditure is essential for achieving the SDG 3.8 targets. It is also essential to establish the nature of the relationship between PHE and OOP.

The body of literature on the determinants of health expenditure has initially focused on the relation between total health care expenditure (THE) and income. This literature is extensive, with a consensus that income is the most important driver of THE [5]. Eventually, the attention of scholars shifted from THE to its components, namely PHE and OOP. PHE is directly influenced by public policy and also by the EU public deficit and debt rules stipulated by the Stability and Growth Pact. Thus, from a public policy perspective, PHE is more relevant to consider than THE. Most of the available literature focusing on PHE is either country-specific or treats PHE as an explanatory variable, rather than a dependent one [6,7]. Similar to THE, the literature on PHE initially focused on macro-economic determinants, mainly GDP, the level of public debt or public deficit and the level of unemployment [8–10]. However, eventually, researchers included various non-macro-economic explanatory variables of PHE. Aging was given particular attention by researchers as they tried to understand whether demographic changes affect PHE [11,12]. Attention was also given to the political influences, such as type of government composition, year in government, political ideologies and years of election [13–15]. Other studies tried to explain differences in PHE by focusing on health system characteristics or by focusing on the level of OOP, in order to identify if OOP and PHE are complimentaries or substitutes [5,16].

This study, based on macro-level EU panel data covering the period from 2000 to 2018, aims to build on the existing knowledge of determinants of PHE by a) studying the relationship between PHE and various macro-economic variables, b) analyzing the relationship between PHE and OOP and c) analyzing how institutional, political and demographic variables effect the level of government health financing. The novelty of this research is that it includes the citizens’ expectations in regards to whether they expect their governments to assume more responsibility, as a possible explanatory variable of PHE.

The six research hypotheses that this study tests are the following:

- A higher level of GDP and level of debt, are associated with higher levels of PHE;
- There is a trade-off between public and private spending on health care: the lower the level of OOP, the higher the level of PHE;
- The more centralized the health system and the tighter the gatekeeping mechanisms, the lower the level of PHE;
- Health care spending reflects political preferences: parties with a leftish political ideology and the election year are positively associated with PHE;
- The aging of the population is a determinant of health care spending: the older the population, the higher the PHE and
- Standards of living and medical innovations raise citizens’ expectations in regards to the level of public healthcare provided. Consequently, if citizens expect governments to assume more responsibility, we should expect a higher level of PHE.

## 2 Literature review

As in the case of THE, macro-economic variables were given particular attention in studies focusing on PHE and differences in these across countries. The income level of a country, as measured by GDP, is a key variable that is commonly applied by researchers in studies focusing on the determinants of PHE. Various studies, such as those by Fan and Savedof [9], Liang and Mirelman [17], and Sfakianakis et al. [18] confirm the positive relationship between PHE and GDP. Although researchers agree that growing incomes increase the demand for health care services and raise health spending, they differ over how much. However, studies focusing on countries with different levels of development, such as Xu et al. [19] and Younsi et al. [20], concluded that the income elasticity is greater than one for the low-income countries, but is smaller than one for lower-middle income, upper-middle income and high-income countries. This demonstrates that the level of PHE depends primarily on a country’s level of economic development. Paes-Sousa et al. argue that in the case of developing countries, such as Brazil, public health expenditure is more sensitive to economic crisis and fiscal austerity policies, than developed countries [21]. Keegan et al. conclude that although PHE is initially resilient to the effects of the economic crises, if such recession is severe, this will eventually have a negative impact on PHE [8]. In contrast, focusing on EU countries, Reeves et al. concluded that economic conditions are not associated with changes in PHE [22]. This study indicates that lower PHE is triggered by more exposure to lending from international financial institutions, reduction in government revenue and budget consolidation decisions. Thus, as public debt increases, governments implement budget consolidation measures, which can involve cutting public expenditure, including that dedicated to healthcare. The negative association between public debt and PHE was also confirmed by other studies, such as that by Lora and Olivera, whose study showed that when public debt increases, social expenditure (including health expenditure) falls [10]. This conclusion is analogous to that of another study focusing on 85 low-and middle-income countries which demonstrated that as the level of public debt increases by 1%, social expenditure falls by around 3% [23].

It is commonly believed that the aging of the population has an impact on PHE. Life expectancy at birth in the EU has increased from 77 to 81 years between the years 2000 and 2019 [24]. Also, the large birth cohorts born after the Second World War have gradually reached an age at which health care use increases. An aging population results in more pressure on health services, due to the increase in age-related diseases and other diseases which are more prevalent among elderly, such as cancer, heart diseases, arthritis, kidney diseases and osteoporosis [25]. Since health care cost per capita increases with age, we expect that the longer the life expectancy, the higher the health expenditure [26]. However, research reveals that there is vast array of interconnected factors that contribute to complexity of the relationship between aging and health expenditure. Some studies have also argued that, irrespective of age, it is the proximity to death that drives PHE. Thus, closer to death, the health cost is expected to increase exponentially [27]. By living a longer life we are actually postponing the death costs. However, such costs tend to be lower for elderly patients, than for younger ones.

The effect of political decisions on health expenditure has also been the topic of debate. Focusing on OECD countries, Herwartz and Theilen conclude that close to elections incumbent governments engage in partisan and opportunistic behavior by increasing PHE in order to increase their reelection probability [15]. This is confirmed by Potrafke who establish, that close to elections, incumbent governments tend to shift their focus to expenditure that produces results in the very short term rather, than on expenditure that produces results in the longer term [28]. In such a situation, social expenditures are given more preference than expenditure on, for example, infrastructural projects, which take longer to become visible for the electorate and will not score any immediate political points. This partisan behavior close to elections is also confirmed by Ferreira et al. [13]. In contrast, focusing on OECD data for the years 1970–2016, Bellido et al. did not find any evidence that incumbent governments increase PHE close to elections [29]. However, they conclude that left-wing governments tended to increase PHE.

The effect of partisan political ideologies on PHE was analysed by various other scholars. Empirical research, such as that published by Potrafke, Ha, and Herwartz and Theilen, contradict the conclusions of Bellido et al. These studies conclude that PHE is not affected by the political ideology of the governing party [14,15,30]. When studying the relationship between the political affiliations of the governing parties or coalitions and social expenditure in 21 OECD countries, Kittel and Obinger found that during the 1980s, the low level of institutional rigidity enabled left and Christian democratic governing parties to increase social expenditure [31]. However, during the 1990s, political ideologies had less impact on social expenditure [29]. This partisan behavior prevailed till 2007. Other studies suggest that political ideology only affects particular government expenditure. For example, in the case of Italy, Russo and Verzichelli found that political ideology only affects expenditure dedicated to defense [32].

The level of gatekeeping at the primary healthcare level and the level of decentralization have also been included by various scholars as possible explanations of PHE differences. Costa-Font and Pons-Novell found that in the case of Spain, between 1992–98, regional institutional differences in the health sector were a key determinant of PHE [33]. The importance of such institutional differences in explaining differences in PHE across EU member states was confirmed by Bech et al. [34]. Primary care or the general practitioner is often the gatekeeper for access to specialized care, hospital care and diagnostic tests [35]. Policymakers believe that the introduction of tighter gate-keeping measures are cost saving and lead to lower PHE. Such cost reductions are achieved by a referral process that prevents unnecessary visits to specialist services, reducing duplicate tests and doctor shopping [36]. The development of this system was triggered by the shortage of specialists and was always considered an efficient measure to control PHE [37].

## 3 Methodology

### 3.1 Data

The data used for this study were extracted from various sources, most importantly from the WHO Global Health Expenditure Database. All the EU member states (*i =* 27) were included in this study and the period covered ranged from year 2000 to 2018 (*t =* 19). The list of the countries included in this study is presented in Table 1. The resulting panel data provided 513 observations.

**Table 1:** List of countries included in this study.

1	Austria	10	France	19	Malta
2	Belgium	11	Germany	20	Netherlands
3	Bulgaria	12	Greece	21	Poland
4	Croatia	13	Hungary	22	Portugal
5	Cyprus	14	Ireland	23	Romania
6	Czechia	15	Italy	24	Slovakia
7	Denmark	16	Latvia	25	Slovenia
8	Estonia	17	Lithuania	26	Spain
9	Finland	18	Luxembourg	27	Sweden

### 3.2 Variables

Similar to Potrafke [14], Behera and Dash [23] and Sfakianakis et al. [18], the dependent variable in this study is the PHE per capita registered by EU member states for the period 2000–2018. The data for this variable have been extracted from the WHO Global Health Expenditure Database [1]. The System of Health Accounts (SHA) manual states that PHE is the summation of seven categories, each having a set of sub-categories [38]. These categories are: (i) curative care, (ii) rehabilitative care, (iii) long-term care (health), (iv) ancillary services, (v) medical goods, (vi) preventive care, and (vii) governance and health system and financing administration.

The explanatory variables included in this study have been chosen for one of the following reasons:

- The variable was found to have a statistically significant effect on PHE or THE in previous studies; or
- The variable was already applied in empirical research focusing on determinants of PHE or THE, but results on its effect were mixed; or
- The variable was never used in empirical research but was hypothesized to be a possible driver of PHE and relevant from a public policy perspective.

The following explanatory variables are included:

**Real GDP Per Capita**—As is customary in most studies focusing on the determinants of PHE we include the size of the national economy, as proxied by real GDP capita, as a determinant of PHE [39].

**Level of Public Debt**—A key variable that can determine a country’s level of PHE is its level of public debt. If debt increases, governments will have more funds available to dedicate to healthcare. In this study, we apply the government consolidated gross debt as a percentage of GDP [39].

**Aging—**Similar to previous literature, such as Steinmann et al. [40], Breyer et al. [41], Bech et al. [34] and von Wyl [42], we include aging as an explanatory variable of PHE. To analyze better the association of aging on PHE, we include to variables in our models: the population aged between 65 and 79 and the population over 80 years [43].

**OOP Expenditure Per Capita**—In cases where the national health systems do not provide the necessary health coverage, patients will have to finance their health needs out of their own pockets [44]. This reliance on OOP expenditure, possibly resulting from a low level of PHE, can ultimately lead to patients facing a barrier to access health care services. In view of the relationship between OOP and PHE, we follow Xu et al. [19] and Halıcı-Tülüce et al. [45], and include OOP one of the determinants of PHE [1].

**Health System Characteristics**—This study looks at two characteristics of health systems that can influence the level of PHE:

- The level of decentralization—Health systems can be distinguished according to whether power is transferred from the central government to regional or local authorities. Since centralized health systems are characterized by central governments retaining most of the control, primarily the budget allocation, it is hypothesized that such health systems result in a lower PHE. Studies on the impact of centralization on PHE are rather limited and mostly focus on only one particular country, such as that published by Costa-Font and Pons-Novell [33], focusing on Spain, and a study focusing on Italy by Guccio and Cavalieri [46]. In this study, we apply the classification of health systems as suggested by the EU Committee of the Regions [47].
- The role of primary health care as gatekeeper—It is believed that the stricter the gatekeeping measures at a primary level of care, the lower the PHE. Inspired by studies such as those by Delnoij et al. [48] and Kringos et al. [35], we include the level of gate-keeping at the primary health care level in our model. Kringos et al. identify four variants of gatekeeping; no gatekeeping, no gatekeeping but incentives exist, partial gatekeeping and full gatekeeping. A dummy variable 1 indicates the existence of a particular variable category, and 0 otherwise [35].

**Values—**Another explanatory variable included in our study captures the opinions of EU citizens regarding whether they expect their respective governments to take more responsibility or if they believe that more responsibility should be taken over by citizens. This indicator, which shows what values EU citizens embrace, was extracted from the European Values Survey [49]. In the model, a dummy variable indicating whether citizens are willing to take more responsibility is valued as 1, while a value of 0 indicates that citizens believe that their governments should assume more responsibility.

**Political Effects**—Finally, following Potrafke [14], Herwartz and Theilen [15] and Russo [32] our study includes political partisan effects on PHE. This is done through the inclusion of two variables that capture partisan effects on PHE, namely political ideologies and the election year. The data regarding these two variables were extracted from the ‘Comparative Political Data Set’[50]. In the case of the variable ‘Election Year’, the dummy variable takes a value of 1 when an election is held and 0 otherwise. With regards to the variable ‘Political Ideology’, a dummy variable is used for left, centre and technocrat governments. In the case of a coalition government, the ideology is based on the political orientation of the party with the highest number of seats of the governing party.

An overview of the sources of the data for our model are presented in Table 2.

**Table 2:** Data sources.

***Dependent Variables***	***Data Source***	***Ref***
Public Health Expenditure per Capita	WHO Global Health Expenditure Database	1
		
***Independent Continuous Variables***	***Data Source***	***Ref***
Real GDP per Capita	Eurostat National Accounts Database	39
Level of Public Debt	Eurostat National Accounts Database	39
Population age 65–79 (as % of total population)	World Population Database	43
Population age 80+ (as % of total population)	World Population Database	43
Out-Of-Pocket Health Expenditure per Capita	WHO Global Health Expenditure Database	1
***Independent Categorical Variables***	***Data Source***	***Ref***
*Time-Invariant*	*** ***	
Level of Decentralization of Health System	EU Committee of Regions Report	47
Role of Primary Health Care as Gatekeeper	Kringos et al.	35
Values	Joint Eureopean Values Survey/World Values Survey	49
*Time-Variant*		
Political Ideology	Comparative Political Data Set	50
Election Year	Comparative Political Data Set	50

### 3.3 Static Econometric models

Once the explanatory variables were identified, we started our analysis using the following static log-log model:

$$logPHE_{it}=\alpha +\beta_{1}logGDP_{it}+\beta_{2}logDEBT_{it}+\beta_{3}logAGE65-79_{it}+\beta_{4}logAGE80{+}_{it}+\beta_{5}logOOP_{it}+\beta_{6}DECENTRALIZATION_{i}+\beta_{7}GATEKEEPING_{i}+\beta_{8}VALUES_{i}+\beta_{9}IDEOLOGY_{it}+\beta_{10}ELECTION_{it}+\nu_{i}+\varepsilon_{it}$$

where PHE is PHE per capita, GDP is GDP per capita, DEBT is the government consolidated gross debt (as at the end of the year) as percentage of GDP, AGE65-79 is the number of inhabitants aged between 65 and 79 percentage of the total population, AGE80+ is the number of inhabitants aged over 80 as percentage of the total population, OOP is the out-of-pocket expenditure per capita, DECENTRALIZATION is the level of (de)centralization in the health care system, GATEKEEPING is the level of gatekeeping at the primary health care, VALUES is whether EU citizens expects governments to provide more or less, IDEOLOGY is the political ideology of the government over the research period and ELECTION is the election year. Finally, *v*_*i*_ is an unobserved country-specific effect; and *ε*_*it*_ is an unobserved white noise disturbance term; i  =  1, …, N (cross-sectional units) and t  =  2, …, T, (time dimensions).

Initially, variables were tested for unit root using the Levin-Lin-Chu test [51], with and without time trends, and for any multicollinearity issues through the application of the Variance Inflation Factor (VIF). Following testing for stationarity and multicollinearity, a number of pooled-OLS, fixed-effects and random-effects regression models were estimated to identify the best model that explains the relationship between PHE and the explanatory variables. The Ramsey RESET test and the Linktest were used to identify any model misspecification in the regression results. The Hausman test was conducted to test the null hypothesis *H*_*0*_ of random-effects against the alternative *H*_*1*_ of fixed-effects and finally, the Breush-Pagan Langrangian Multiplier Test was also applied to identify any cross-sectional dependence in the panel data.

In regard the categorical explanatory variables, for the purpose of conducting the regressions, the largest category was used as the reference category. In the case of the fixed-county effects model, the reference country used was Germany. Tables 3 and 4 provide descriptive statistics on the explanatory variables for years 2000 and 2018.

**Table 3:** Descriptive statistics—continuous variables.

Variables	Description	Number of	Mean	Standard	Minimum	Maximum
		Obs		Deviation		
logPHEit	Government schemes and compulsory contributory health care financing schemes in current PPP per capita	513	3.198465	0.303497	2.297214	3.717908
logGDPit	GDP per capita	511	4.285089	0.299245	3.47567	4.94507
logOOPit	Household out-of-pocket payments in current PPP per capita	513	2.637478	0.255112	1.67923	3.13696
logPublicDebtit	Consolidated gross debt as percentage of GDP	513	1.670064	0.311974	0.579784	2.26998
log65-79it	Population aged between 65–79 as percentage of the total population	513	1.094543	0.062441	0.911212	1.21009
log80+it	population over 80 and above as percentage of the total population	513	0.58459	0.121568	0.241683	0.842332

**Table 4:** Descriptive statistics—categorical variables.

Variables	Description	Year of	Categories	Number of	%
* *	* *	Observation	* *	Observations	
**Time Invariant Variables**					
					
Decentralization	The level of health system decentralization	2017	Operatively Decentralized Health System^	9	33.33%
			Partially Decentralized Health System	6	22.22%
			Decentralized Health System	4	14.81%
			Mostly Centralized Health System	3	11.11%
			Centralized Health System	5	18.52%
					
Gatekeeping	The level of health system decentralization	2015	Full Gatekeeping^	10	37.04%
			No Gatekeeping But Incentives Exit	7	25.93%
			Partial Gatekeeping	4	14.81%
			No Gatekeeping	6	22.22%
					
Values	Individual vs State responsibility for providing	2017*	Individuals Should Provide More	17	62.96%
			State Should Provide More	10	37.04%
**Time Variant Variables**					
** **	** **	** **	** **	** **	** **
Ideology	Political ideology of the governing party	2000–18	Centre governments *(nr of years)*	105	20.47%
** **	** **	** **	Left-wing governments *(nr of years)*	182	35.48%
** **	** **	** **	Right-wing governments *(nr of years)*	224	43.66%
** **		** **	Technocrat governments *(nr of years)*	2	0.39%
					
Election	Year of government election	2000–18	Election year	134	26.12%
			Not an election year	379	73.88%

^ Applied as reference category.

*Data for Belgium, Estonia, Ireland, Luxembourg, Malta and Poland is based on 2008.

### 3.4 Dynamic econometric model

A shortcoming of the fixed-effects and random-effects regression models is that these do not address the issue of endogeneity. To address dynamic endogeneity two methods are frequently used. The first method is the difference-Generalized Method of Moments (GMM) developed by Arellano and Bond [52], which corrects for endogeneity by transforming all regressors into first differences. However, lagged levels are frequently found to be poor instruments for first differences [53]. Another shortcoming of the difference-GMM is that time-invariant variables cannot be included as explanatory variables. In contrast, the two-step system GMM proposed by Arellano and Bover [53] and Blundell and Bond [54] applies lagged values and internal transformations to address the problem of endogeneity [55]. System GMM transforms the model into two equations; one expressed in levels with first differences as instruments, while another is expressed in a first difference form with levels as instruments. This method generates more reliable and consistent estimators than the difference GMM and allows the use of time-invariant explanatory variables. Following, Xu et al. [19], Atems [56] and Sfakianakis et al. [18], this study applies system GMM.

Since a large number of instruments can result in over-identification, we follow Roodman (2009) and limit the number of instruments in our estimation [57]. This is done by limiting the number of lags of the predetermined variables used as instruments to two, rather than using all the available lags. We also apply the widely used Windmeijer (2005) robust standard errors, which corrects the bias arising from using the efficient weight matrix being evaluated at an estimate, instead of the true value [58]. The Sargan J-statistic (1958) is applied to test overidentifying restrictions [59]. To test the absence of second-order serial correlation the Arellano-Bond (1991) is applied. The Arellano–Bover/Blundell–Bond’s dynamic panel data estimators were found by applying the ‘xtabond2’ STATA syntax developed by Roodman (2009) [57].

The dynamic model specification when applying the system-GMM regression is given as follows:

$$logPHE_{it}=\beta_{1}logPHE_{it-1}+\beta_{2}logGDP_{it}+\beta_{3}logDEBT_{it}+\beta_{4}logAGE65-79_{it}+\beta_{5}logAGE80{+}_{it}+\beta_{6}logOOP_{it}+\beta_{7}DECENTRLIZATION_{i}+\beta_{8}logGATEKEEPING_{it}+\beta_{9}VALUES_{i}+\beta_{10}IDEOLOGY_{it}+\beta_{11}ELECTION_{it}+\varepsilon_{it}$$

## 4 Results

### 4.1 Static regression results

The results of the Levin-Lin-Chu tests, presented in S1 Appendix, reject the unit root null hypotheses and thus provide comfort with regard to the stationarity of the continuous variables used in the model. The VIF test indicated that there is no multicollinearity issue, while the Ramsey RESET test and the Linktest confirmed that there were no misspecifications in the static regression models. Furthermore, since the Hausman test showed that the differences in coefficients were not systematic, the random-effects model was considered more efficient and consistent than the fixed-effects model. However, since no cross-sectional dependence was identified through the application of the Breush-Pagan Langrangian Multiplier Test, it was concluded that the pooled-OLS model provides the best estimation of the relationship between PHE and the dependent variables.

In this study, two static regression models are presented: the first one is the random-effects model, while the second is the fixed-country effects model. In the latter model, the country-specific time-invariant explanatory variables (which were included in the random-effects model) were removed and replaced by the country dummy variable. The country variable captures cross-sectional heterogeneity in regard to all time-invariant factors across EU member states. The static regression results are presented in Models 1 and 2 in Table 5.

**Table 5:** Regression results.

			Model 1		Model 2		Model 3	
Explanatory Variable	Categories	Dummy	Dependent Variable—logPHE					
		Variable	Random-Effects GLS Regression		GLS Fixed Country Effects		Two-Step System GMM Regression	
			Coefficient	Std Error	Coefficient	Std Error	Coefficient	Std Error
** **	** **	** **	** **	** **	** **		** **	** **
logPHE_it-1_	-	-	-	-	-	-	0.706***	(0.086)
logGDP_it_	-	-	0.659***	(0.141)	0.555***	(0.176)	0.167*	(0.088)
logOOP_it_	-	-	0.386***	(0.093)	0.413***	(0.105)	0.205***	(0.069)
logPublicDebt_it_	-	-	0.071**	(0.030)	0.051	(0.035)	0.013	(0.025)
log65-79_it_	-	-	0.364	(0.228)	0.398	(0.245)	0.056	(0.092)
log80+_it_	-	-	0.358***	(0.119)	0.388***	(0.133)	-0.042	(0.100)
Ideology	(Right)^	-	-	-	-	-	-	-
	Centre	-	0.000	(0.012)	-0.002	(0.012)	0.007	(0.010)
	Left	-	-0.007	(0.007)	-0.008	(0.007)	0.003	(0.005)
	Technocrat	-	-0.001	(0.019)	-0.004	(0.019)	0.013	(0.012)
Decentralization	(Operatively Decentralized Health System)^	-	-	-	-	-	-	
	Decentralized Health System	-	0.28***	(0.069)	-	-	0.112***	(0.033)
	Partially Decentralized Health System	-	0.201***	(0.067)	-	-	0.084**	(0.035)
	Mostly Centralized Health System	-	0.158***	(0.059)	-	-	0.048	(0.030)
	Centralized Health System	-	0.259***	(0.037)	-	-	0.097***	(0.031)
Gatekeeping	(Full Gatekeeping)^	-	-	-	-	-	-	-
	No Gatekeeping	-	-0.139**	(0.055)	-	-	-0.069***	(0.018)
	No Gatekeeping But Incentives Exit	-	0.033***	(0.045)	-	-	-0.008	(0.018)
	Partial Gatekeeping	-	-0.112***	(0.043)	-	-	-0.035***	(0.012)
Election	Election	1	-0.003	(0.002)	0.003	0.0024563	0.004**	(0.002)
	No Election	0	-	-	-	-	-	-
Values	Individuals Should Provide More	1	-0.016	(0.035)	-	-	-0.011	(0.012)
	State Should Provide More	0	-	-	-	-	-	-
EU Member State	(Germany)^	-	-	-	-	-	-	-
	Austria	-	-	-	-0.077***	(0.026)	-	-
	Belgium	-	-	-	-0.11***	(0.026)	-	-
	Bulgaria	-	-	-	-0.207	(0.143)	-	-
	Croatia	-	-	-	0.07	(0.062)	-	-
	Cyprus	-	-	-	-0.458***	(0.071)	-	-
	Czechia	-	-	-	0.118***	(0.045)	-	-
	Denmark	-	-	-	-0.02	(0.031)	-	-
	Estonia	-	-	-	-0.082	(0.084)	-	-
	Finland	-	-	-	-0.119***	(0.018)	-	-
	France	-	-	-	0.075***	(0.027)	-	-
	Greece	-	-	-	-0.33***	(0.047)	-	-
	Hungary	-	-	-	-0.108	(0.077)	-	-
	Ireland	-	-	-	0.091	(0.061)	-	-
	Italy	-	-	-	-0.206***	(0.018)	-	-
	Latvia	-	-	-	-0.323***	(0.098)	-	-
	Lithuania	-	-	-	-0.132	(0.092)	-	-
	Luxembourg	-	-	-	0.021	(0.069)	-	-
	Malta	-	-	-	-0.119*	(0.066)	-	-
	Netherlands	-	-	-	0.08***	(0.030)	-	-
	Poland	-	-	-	-0.05	(0.083)	-	-
	Portugal	-	-	-	-0.162***	(0.051)	-	-
	Romania	-	-	-	0.016	(0.099)	-	-
	Slovakia	-	-	-	0.085	(0.064)	-	-
	Slovenia	-	-	-	0.046	(0.035)	-	-
	Spain	-	-	-	-0.149***	(0.032)	-	-
	Sweden	-	-	-	-0.065***	(0.019)	-	-
Constant	-	-	-1.486***	(0.569)	-0.938	(0.700)	-0.397***	(0.342)
Observations			511		511		485	
Wald chi-square/(prob.)			1,277.67/(0.000)		**-**		2,220,000/(0.000)	
R-squared (within)			0.896		0.897		**-**	
R-squared (between)			0.924		1.000		**-**	
R-squared (overall)			0.918		0.978		**-**	
AR (1)/(prob.)			**-**		**-**		-2.45/(0.014)	
AR (2)/(prob.)			**-**		**-**		0.40/(0.688)	
J-Statistic / (prob.)			**-**		**-**		3.00/(0.392)	

^ Applied as reference category.

***, ** and * denote statistical significance at the 1%, 5% and 10% levels, respectively.

### 4.2 Dynamic regression results

The Sargan test confirms that there are no over-identifying restrictions. The diagnostic test applied to test for serial correlation, namely the Arellano-Bond (1991) test, indicate the presence of first order serial correlation, but no ***second*** order correlation in the error terms. This confirms that the time-lag applied in our model is valid and that it is not necessary to apply deeper lags. The system GMM regression results are presented in Model 3, in Table 5.

### 4.3 Analysis of results

In both the static models, the strongest statistically significant explanatory variable of PHE per capita results to be GDP per capita. Applying these models, a change of 10% in GDP per capita, results in an increase of PHE amounting to 6.6%, while applying the dynamic model the impact is reduced to 1.7%. When applying the GMM dynamic model, OOP per capita results to be the strongest statistically significant variable. Results indicate that OOP and PHE are complements rather than substitutes: if PHE increases by 10%, OOP increases by 2%. The level of public debt results to be associated with PHE, but only when applying the static models.

Results also indicate that 14 EU member states have time-invariant variables that significantly affect PHE, at the 10% confidence level. However, such effects were rather modest for most of these member states, with the exception of Latvia, Italy and Cyprus. The GMM results also show that PHE tends to be modestly affected by election years, while the static regression results indicate that a higher public debt is associated with higher level of PHE.

Furthermore, health system characteristics, represented by the level of gatekeeping and decentralization, result to be associated with PHE, with the association between gatekeeping and PHE being more modest than that with the level of decentralization. In contrast, the political orientation of the governing party, the aging population and citizens’ expectations, proxied by the variable ‘*values*’, do not result to be associated with PHE.

## 5 Discussion

Using panel data covering 18 years, we studied the key determinants of PHE in EU member states, using both static and dynamic regression models. As expected, a strong statistically significant positive relationship was identified between GDP per capita and PHE per capita. This is consistent with earlier findings in the literature on this relationship. In view of this, we conclude that governments adjust their PHE depending on the economic situation. A 10% change in GDP per capita is associated with a change amounting to 6% in PHE, when applying the pooled-OLS model, and 5.5% when applying the fixed-country effects model. This association drops to 2% when applying the dynamic model. Similar to Xu et al., the static models identify a higher income elasticity when compared to the results generated by the dynamic models. However, the coefficients of our results are more conservative than those of Xu et al. Thus, these results confirm that as the economies of the EU member states grow, PHE per capita is also expected to increase. This conclusion is consistent to those of Akca et al. [7], Xu et al. [19] Behera and Dash [23] and Sfakianakis [18]. Halıcı-Tülüce et al. find that in high-income countries, in the short-term there is a two-way causality between PHE and GDP [45]. Consequently, as health expenditure increases we can expect a positive effect on PHE and vice-versa. In contrast, Keegan et al., focusing on the effect of the 2009 recession on EU member states, found that PHE, together with OOP and THE, were resilient to the effects of the crisis [8].

The other macro-economic variable included in this study, namely the level of public debt, appears to be associated with PHE, only when we apply the static models. The positive association between PHE and GDP results to be rather modest and is significant only the 5% level of confidence. Thus, if the public debt level increases PHE also increases. This conclusion is consistent to the results of empirical research, such as that by Chang et al, who focus on 13 OECD countries, found a strong a positive correlation between social expenditure, including PHE, and public debt [60]. This relationship appeared to be stronger in the short run, rather than in the long run. Using data covering 120 countries for the years 1995 and 2010, Liang and Mirelman, also found a positive relationship between government debt and PHE [17]. Studies focusing on countries with a lower level of GDP tend to identify a negative relationship between the debt level and PHE. Focusing on 85 low- and middle-income countries, Behera and Dash found that the fiscal deficit and the debt services payment have a negative effect on PHE in low-and middle-income countries. Mahdavi, who focused on data of 47 developing countries covering 1972–2001, indicates that as the debt burden increases, governments change the composition of their spending, which results in social expenditure being adversely impacted. This conclusion is shared by Fosu, who limited their analysis to 35 African countries, and Lora and Olivera, who studied the relationship between public debt and social expenditure using an unbalanced panel of 50 developing countries [10,61].

Results from both the static and dynamic models, also indicate that OOP and PHE are complements rather than substitutes, The static model indicates that a 10% increase in PHE is associated with a 4% increase in OOP health expenditure per capita. Again, the dynamic regression generated more conservative results, with the 10% increase in PHE per capita being associated with a 2% increase in OOP health expenditure per capita. Results from empirical research focusing on this association are mixed. Guccio and Cavalieri establish that in the case of Italy, PHE and OOP health expenditure are complements in higher-income regions and are substitutes in lower-income ones [46]. Focusing on EU member states, Grima et al. conclude that PHE and OOP are complements [62]. Bhattacharya and Qiao also suggest that investments in the private health sector are more productive if complemented by PHE [3]. In contrast, when examining the association between the lagged effect of OOP health expenditure on PHE, Tuohy et al. found a negative and significant relationship [31]. However, these academics argue that the dynamic nature of the relationship between public and private health finance depends on various factors, with the most important one being each country’s boundary between the public health sector and the private one.

Such disagreement on the significance of the association between OOP expenditure and PHE leads to a cautious position when making inferences on these variables. In 2018, the highest OOP health expenditure was registered by Malta, followed by Cyprus and other Eastern European and Mediterranean countries. Grima et al. suggest that the higher OOP health expenditure in Mediterranean countries can be attributed to the fact that patients prefer to have a one-to-one relationship with clinicians [62]. However, higher OOP health expenditure can also be caused by lower-than-average PHE expenditure, especially in the case of Eastern European countries. A low level of PHE expenditure results in poorer health services and longer waiting times. Consequently, patients in these countries resort to the private sector to meet their health needs [63].

Removing gatekeeping measures at the primary level of health results in a reduction in PHE. Evidence on the effectiveness of gatekeeping to control PHE is not as straightforward as politicians might suggest. The introduction of gatekeeping measures may result in higher costs due to generous incentives offered to doctors to act as gatekeepers, which ultimately reduce the expected level of savings [26]. In contrast, Garrido et al. observed that the majority of empirical research shows that gatekeeping is an effective cost-containment mechanism [31]. They argue that gatekeeping results in a lower level of health services utilization, which consequently leads to lower health expenditure. Such a perspective is also shared by Gerdtham et al. [37]. Focusing on 18 OECD countries, Delnoij et al. [36] conclude that although gatekeeping is expected to reduce ambulatory costs and costs regarding the provision of outpatient health services, overall, THE is not expected to be impacted. Although diverging views exist on the importance of gatekeeping initiatives as cost-containment measures, it should be noted that such measures may also be introduced to improve governance at the primary health care level or to enhance the coordination between the primary and secondary levels of health care [35]. However, Kringos et al. argue that a stronger primary health care service is associated with lower socioeconomic health inequalities, lower avoidable hospitalizations and better health outcomes [35].

Regression results also reveal that the level of centralization is associated with the level of PHE. Results indicate that decentralized public health systems have a modestly higher positive association with PHE, when compared to centralized systems. This can be related to the fact that in the latter systems, the central government retains the power, responsibility and functions related to the delivery of the health service. Research focusing on the Spanish decentralized health model also reveals that regions with fiscal and political responsibilities have a higher PHE than those without such responsibilities [33]. López Casasnovas and Saez [64] reached the same conclusion when doing an empirical study on 110 regions in 8 OECD countries. Alves et al. argue that this higher expenditure may result from duplication of inputs, diseconomies of scale or may be caused by sunk implementation costs of such decentralized systems [63]. Focusing on the Italian regional health system, Guccio and Cavalieri confirmed that decentralization led to higher-income regions having a higher share of PHE, ultimately resulting in regional inequalities [46]. In such scenario, governments of member states must intervene so that regional health expenditure disparities do not persist.

Our results also establish that political ideologies have no statistically significant association with PHE. Empirical research, such as that published by Potrafke, Herwartz and Theilen, and Ha, also confirm that PHE is not affected by the political ideology of the governing party [14,15,30]. When studying the relationship between the political affiliations of the governing parties or coalitions and social expenditure in 21 OECD countries, Kittel and Obinger found that during the 1980s, the low level of institutional rigidity enabled left and Christian democratic governing parties to increase social expenditure [33]. However, during the 1990s, political ideologies had less impact on social expenditure. Bellido et al., in an analysis of OECD data for the years 1970–2016, concluded that left-wing governments tended to increase PHE [29]. This partisan behavior prevailed till the financial crisis of 2007. Bellido et al. also conclude that PHE is higher when there are long-lasting governments, minority governments and governments with a higher legislative power. Other studies suggest that political ideology only affects particular government expenditure. For example, in the case of Italy, Russo and Verzichelli found that political ideology only affects expenditure dedicated to defense [32].

Contrary to political ideologies, the election year has a positive, but very modest, association with the level of PHE. This suggests that incumbent governments tend to increase health expenditure during election years. The partisan effect resulting from elections was also identified in empirical research. Potrafke established that in election years, incumbent governments tend to shift their focus to expenditure that produces results in the very short term rather than on expenditure that produces results in the longer term [44]. Thus, in such a situation, social expenditures are given more preference than expenditure on infrastructural projects, which will not score any immediate political points. Once again, our conclusion contrasts to that of Bellido et al., with this study finding no evidence that incumbent governments engage in an opportunistic behavior by increasing PHE close to elections [29].

In regards aging, when applying the dynamic models, both age cohort variables included in this study do not have an association with PHE. These results are consistent with the literature focusing on the impact of longevity on health expenditure. Various studies suggest that the actual driver of health expenditure is not age itself, but the proximity to death [11]. This theory was incepted by Zweifel et al. in their seminal paper in which they applied time to death as an explanatory variable to health expenditure [65]. They argue that when policymakers believe that aging is a driver of health expenditure, they will be diverting their attention from the actual determinants. This argument, labelled as the ‘red herring’, was confirmed by subsequent studies. Rechel et al. argue that the high costs associated with older people are caused by the fact that these are more likely to die within a year, when compared to younger people [27]. Recent studies by Raitano and Williams et al. also confirm that aging is not a primary driver of health expenditure [11, 12].

Focusing on Germany, Brockmann suggest that the relationship between PHE and aging is negative due to the fact that older patients receive less intensive and expensive treatment and the fact that doctors may prefer to give more attention to younger patients, whose life expectancy is higher [66]. Thus, the cost of dying at a relatively younger age is higher than at an older age. Vinkel Hansen et al. concluded that the main determinant of health expenditure is the number of chronic diseases [67]. Von Wyl argues that to make more correct PHE projections, there should be a better understanding of the relationship between aging and the prevalence of chronic diseases [42]. The J-shaped curve between age and health expenditure has been confirmed by other studies, such as that by Gabriele et al. [26]. However, as is the case for other variables applied our research, there are studies that provide a different conclusion, such as those of Behera and Dash [23] and Sfakianakis et al. [18].

With regards to the relationship between unidentifiable country specific effects and PHE, compared to Germany, our regression results identify Czechia, the Netherlands and France as the countries in which the fixed-country effects are positively associated with the level of PHE. In 2018, these three particular countries were amongst those with the highest PHE when as percentage of THE [3]. In the Netherlands, PHE expressed as percentage of GDP, was already above average in 2000. This figure increased from 5.32% in 2000 to 8.24% in 2018. This was the third-highest increase in PHE/GDP in the EU between 2000 and 2018. Bakx et al. argue that the increase in PHE is triggered by greater hospital use and the introduction of new technologies [68].

The fixed-country effects regression results also identify eleven countries with time-invariant country-specific factors being negatively associated with PHE. Cyprus and Greece resulted to have the strongest negative association with PHE. In the case of Cyprus, between 2012 and 2015, government introduced PHE cuts and restricted health coverage. Thus, access to health care and financial protection deteriorated [69]. Similarly, the Greek public health service experienced a substantial reduction in its financing, which was imposed by the EU, following the 2009 financial crisis. These reductions were part of bailout conditions required to reduce Greece’s overall government expenditure. The austerity measures introduced between 2009 and 2012, triggered Greece’s PHE to decrease by 25.2% [70]. Between 2008 and 2018, the rate of PHE as percentage of THE fell from 65.33% to 58.75% [1].

In summary, we accept the hypotheses that the level of GDP, the debt level, and the election year are positively associated with PHE. We also accept the hypothesis that a decentralized health system leads to higher PHE. However, we reject the hypothesis that there is a negative association between OOP and PHE, and that political ideologies, citizens’ expectations and the amount of aging population affect PHE. Finally, we also reject the hypothesis that stricter gatekeeping measures at the primary level of the health care result in lower PHE.

Before concluding, it is important to note a few limitations to this study. First, economic events, such as recessions and the 2008–09 economic crisis, may affect the generalizability of our results. Second, the variables included in this study are rather limited when compared to the various complexities surrounding the provision of public health care, such as cross-country variations in prices and various other political and socioeconomic factors. In this study, such unobserved explanatory variables were captured in the fixed-country effects model. Nevertheless, the study still adds to our knowledge on the key drivers of PHE in the EU, particularly on the differences in health systems. Future research can focus more on the drivers of the sub-categories of PHE, particularly the expenditure on preventive care, which the COVID-19 pandemic showed is necessary to have resilient health systems in the face of health crises.

## 6 Conclusion

This paper examined the relationship between the level of PHE and various explanatory variables in EU member states during the years 2000 to 2018, with the aim of identifying key drivers of PHE expenditure. Our analysis reveals that GDP and OOP are the predominant drivers of PHE. Other non-income variables, namely the election year, the level of public debt, the level of gatekeeping at primary health care level and centralization of the health system, only exerting a modest effect on PHE. These results are largely consistent with the findings of studies examining the determinants of both PHE and THE, focusing on high-income countries. Furthermore, our study also indicates that country-specific effects exert only a minor influence on PHE.

During the last two decades, various EU member states have largely relied on OOP expenditure. This makes it more difficult for these countries to achieve universal health coverage, which is one of the 17 Sustainable Development Goals adopted by the United Nations aimed at achieving decent lives for all by year 2030. Such high dependence on private funding, imposes a substantial financial burden on households, especially the lower-income ones and consequently can result in furthering health inequalities.

Another important policy implication is that reforms to health systems, such as implementing tighter or more relaxed gatekeeping controls, should be introduced with the aim of offering better health service to patients, rather than to control health expenditure. Thus, before introducing such reforms, more evidence is needed on the effects on patients, as ultimately patients may end up receiving a worse health service.

Finally, due to the fact that sustaining increases in PHE largely depends on stronger economies, during periods of economic challenges, policymakers must implement more policies that promote the more efficient use of available resources. Such policies may include the introduction of Health Technology Assessments, improvements in procurement and operational efficiency, increase in day surgeries and reducing low-value care and unnecessary interventions. In view of the importance of efficiency in health systems, especially in the post-pandemic era, EU must also give more emphasis to such indicators rather than just giving importance to input and output indicators, such as PHE per capita and life expectancy, respectively.

## Supporting information

S1 Appendix Levin-Lin-Chu Unit-Root Tests. (DOCX)

S2 Appendix Dataset. (XLSX)
