# Supplementary material for: Determinants of public health expenditure in the EU
Source: PLoS One. 2024 Mar 6;19(3):e0299359. doi: 10.1371/journal.pone.0299359 (PMC10917289; doi:10.1371/journal.pone.0299359)
Supplement: S1 Appendix — (DOCX) [file pone.0299359.s001.docx]

| ***S1 Appendix - Levin-Lin-Chu Unit-Root Tests*** | | | | | | | | | | | | | | | | | | | |
| --- | --- | --- | --- | --- | --- | --- | --- | --- | --- | --- | --- | --- | --- | --- | --- | --- | --- | --- | --- |
|  |  |  |  |  |  |  |  |  |  |  |  |  |  |  |  |  |  |  |  |
|  | **Lags (Year)** |  | **logPHE** | |  | **logGDP** | |  | **logPublicDebt** | |  | **log65-79** | |  | **log80+** | |  | **logOOP** | |
|  |  |  | **Statistic** | **p-value** |  | **Statistic** | **p-value** |  | **Statistic** | **p-value** |  | **Statistic** | **p-value** |  | **Statistic** | **p-value** |  | **Statistic** | **p-value** |
| NoTime Trend | 0 |  | -6.259 | 0.000 |  | -2.472 | 0.007 |  | -3.641 | 0.000 |  | -1.885 | 0.030 |  | -12.740 | 0.000 |  | -12.093 | 0.000 |
|  | 1 |  | -6.259 | 0.000 |  | -2.472 | 0.007 |  | -3.641 | 0.000 |  | -1.885 | 0.030 |  | -12.740 | 0.000 |  | -12.093 | 0.000 |
| With time trend | 0 |  | -4.652 | 0.000 |  | -4.276 | 0.000 |  | -2.450 | 0.932 |  | -3.961 | 0.932 |  | -4.709 | 0.000 |  | -10.474 | 0.000 |
|  | 1 |  | -4.652 | 0.000 |  | -4.276 | 0.000 |  | -2.450 | 0.932 |  | -3.961 | 0.932 |  | -4.709 | 0.000 |  | -10.474 | 0.000 |
